# Who is accessing public-sector anti-retroviral treatment in the Free State, South Africa? An exploratory study of the first three years of programme implementation

**DOI:** 10.1186/1471-2458-10-387

**Published:** 2010-07-01

**Authors:** Edwin Wouters, Christo Heunis, Koen Ponnet, Francis Van Loon, Frederik le Roux Booysen, Dingie van Rensburg, Herman Meulemans

**Affiliations:** 1 Department of Sociology and Research Centre for Longitudinal and Life Course Studies, University of Antwerp, Sint-Jacob Street 2, 2000 Antwerp, Belgium; 2 Centre for Health Systems Research and Development, University of the Free State, Nelson Mandela Avenue, Bloemfontein, South Africa; 3 Department of Economics, University of the Free State, Nelson Mandela Avenue, Bloemfontein, South Africa

## Abstract

**Background:**

Although South Africa has the largest public-sector anti-retroviral treatment (ART) programme in the world, anti-retroviral coverage in adults was only 40.2% in 2008. However, longitudinal studies of who is accessing the South African public-sector ART programme are scarce. This study therefore had one main research question: who is accessing public-sector ART in the Free State Province, South Africa? The study aimed to extend the current literature by investigating, in a quantitative manner and using a longitudinal study design, the participants enrolled in the public-sector ART programme in the period 2004-2006 in the Free State Province of South Africa.

**Methods:**

Differences in the demographic (age, sex, population group and marital status) socio-economic (education, income, neo-material indicators), geographic (travel costs, relocation for ART), and medical characteristics (CD4, viral load, time since first diagnosis, treatment status) among 912 patients enrolled in the Free State public-sector ART programme between 2004 and 2006 were assessed with one-way analysis of variance, Bonferroni post-hoc analysis, and cross tabulations with the chi square test.

**Results:**

The patients accessing treatment tended to be female (71.1%) and unemployed (83.4%). However, although relatively poor, those most likely to access ART services were not the most impoverished patients. The proportion of female patients increased (*P *< 0.05) and their socio-economic situation improved between 2004 and 2006 (*P *< 0.05). The increasing mean transport cost (*P *< 0.05) to visit the facility is worrying, because this cost is an important barrier to ART uptake and adherence. Encouragingly, the study results revealed that the interval between the first HIV-positive diagnosis and ART initiation decreased steadily over time (*P *< 0.05). This was also reflected in the increasing baseline CD4 cell count at ART initiation (*P *< 0.05).

**Conclusions:**

Our analysis showed significant changes in the demographic, socio-economic, geographic, and medical characteristics of the patients during the first three years of the programme. Knowledge of the characteristics of these patients can assist policy makers in developing measures to retain them in care. The information reported here can also be usefully applied to target patient groups that are currently not reached in the implementation of the ART programme.

## Background

In November 2003, the South African Cabinet announced the *Operational Plan for Comprehensive HIV and AIDS Care, Management and Treatment for South Africa *[1]. This policy document set out a coherent, comprehensive strategy to deal with human immunodeficiency virus (HIV) and acquired immunodeficiency syndrome (AIDS) and explicitly included the use of anti-retroviral medicines for the treatment of HIV and AIDS (anti-retroviral treatment or ART) for HIV/AIDS patients with CD4 count ≤ 200 cells/μl or World Health Organization (WHO) stage IV disease [2]. In 2007, the *HIV & AIDS and STI National Strategic Plan (NSP) 2007-2011 *re-affirmed this emphasis on treatment by adopting as its two primary aims to reduce new HIV infections by 50%, particularly through the prevention of mother-to-child transmission (PMTCT), and to reduce the impact of the epidemic by extending access to appropriate treatment, care, and support to 80% of people diagnosed with HIV [3].

Preliminary research results showed that ART provided by the South African government health services is as effective as that provided in high-income countries [4]. The magnitude of the improvements in health and quality of life attributable to ART is similar to the estimates for non-governmental organisations (NGOs) and research-supported ART services in developing countries and for routine treatments in Europe and the United States [4-10]. Studies of the ART programme outcomes have also indicated that South Africa ranks among the top African countries in patient survival and retention rates [11,12]. Finally, despite severe human resource shortages, health personnel provide high-quality care, which results in high levels of patient satisfaction with ART-related services [13-15].

These favourable treatment outcomes should not encourage complacency. Despite having the largest public-sector ART programme in the world, the South African ART roll-out is hampered by limited financial and human resources [16-18]. The health-care system is facing a crisis and the government is falling behind in its efforts to meet the *NSP *targets, including the treatment of 80% of people who require ART by 2011 [3]. By the middle of 2008, 568,000 HIV-infected patients were receiving ART in South Africa, with the public health sector accounting for 79% of this total. Based on the current Department of Health criteria for defining anti-retroviral eligibility (CD4^+ ^count < 200/μL or World Health Organization [WHO] stage IV), anti-retroviral coverage in adults was only 40.2% in 2008 [19]. Moreover, coverage varied significantly between the provinces, with the Free State, the subject of this study, ranking last (25.8%)[19]. This creates a risk of ART facilities becoming "islands of excellence in a sea of under provision" [20-22]. ART facilities have a positive impact on the lives of patients enrolled in the programme, but after five years of treatment roll-out, their effects are still inequitable, serving less than half of the people who currently require ART in South Africa [23]. The large gap between the number of people eligible for ART and the number of people actually accessing care creates a nationwide problem, with long waiting lists. Recent studies by Jacobs et al. (2008) and Rosen et al. (2005) have shown that the large patient numbers and resource constraints mean that ART in South Africa will be rationed for many years to come [24,25].

Bennet and Chanfreau (2005) have stressed that it is crucial to monitor who has access to treatment when anti-retroviral programmes are implemented in regions in which they are most needed [26]. Knowing the characteristics of the enrolled patients can potentially facilitate the successful implementation of the *NSP *in two ways. First, knowledge of the characteristics of the enrolled patients can assist policy makers in developing measures to retain these patients in care, a necessary condition for every ART programme. Second, information about these patients' characteristics can identify explicit and implicit rationing systems when the over- and under-represented groups of patients are evaluated [27]. In this respect, Rosen et al. (2005) identified different types of rationing systems, using socio-economic, geographic, and medical criteria [25]. The information produced could then be usefully applied by policy makers to target patient groups that are not reached in the implementation of the current ART programme.

Longitudinal studies of who is actually accessing the South African public-sector ART programme are scarce [28]. This study has therefore one main research question: who is accessing public-sector ART in the Free State Province of South Africa? The study aimed to extend the current literature by investigating, in a quantitative manner and using a longitudinal study design, the participants enrolled in the public-sector ART programme in the period 2004-2006 in the Free State Province of South Africa. Interview data for 912 ART patients were used to assess the differences in the demographic (age, sex, population group, marital status), socio-economic (education, income, neo-material indicators), geographic (travel costs, relocation to obtain ART), and medical (CD4^+ ^count, viral load, time since first diagnosis, treatment status) characteristics between patients enrolled at various points in time. Furthermore, by identifying differences between the patients who enrolled at different time points, the study assessed whether certain patient groups were benefitting disproportionately from the public-sector ART programme, so that the distribution of public health benefits was uneven across the population. This information could be usefully applied in the development of policy measures that target groups of eligible patients who are not currently accessing ART.

## Methods

### Setting

This study is part of a longitudinal study (entitled *Public sector anti-retroviral treatment: documenting, monitoring, evaluating, and facilitating implementation of the national treatment plan in the Free State Province, South Africa*) of patients enrolled in the public-sector ART programme in the Free State Province of South Africa. This research was approved by the Ethics Committee of the Faculty of Humanities, University of the Free State, and authorised by the provincial Department of Health.

### Study population

The larger longitudinal study had two distinct objectives. Firstly, the patient survey needed to generate data that could be employed to investigate the experience over time of a representative cohort of patients that qualify for ART at the start of the public-sector ART programme in the Free State (i.e. CD4 < 200 cells/μL and/or WHO stage IV AIDS). It was estimated, based on the ASSA2000 model, that a total of 28 290 HIV-positive persons would develop WHO stage IV AIDS defining illness annually in the province [29]. In order to detect a 5% difference in this population at the 95% confidence level, one needed to observe a sample of 379 patients. However, because estimates of the number of patients with WHO stage IV AIDS defining illness were not available at the district level, it was decided not to sample patients from each district in proportion to population size and HIV-prevalence in the particular district, but rather to sample an equal number of patients in each district. In light of available resources, the estimated sample size of 379 was inflated to 400, with 80 patients to be interviewed per district. In cases where the list included fewer than 80 patients, as in Xhariep District, a census of all treatment and non-treatment cases was conducted. This resulted in a total sample size of 371 patients interviewed in the first months after the start of the ART programme. The Free State Department of Health implemented ART in a phased or staggered manner: the first district (Lejweleputswa) started in February 2004, the last district (Fezile Dabi) only started in December 2004. The current study only employs this baseline data. However this *cohort *of 371 patients was followed over the entire three-year period.

Secondly, the patient survey also needed to generate data that could be employed to generalise about the experiences of patients that were in the ART programme in each of the five districts in the Free State Province as well as in the province as a whole over a three-year period (2004-2006). The objective here was to provide a representative picture of patient experiences over the course of the rollout of ART in the province and each of its five districts, so as to evaluate the treatment programme in terms of select outcomes. However, data from the interviews with cohort patients could not be used to draw conclusions about the treatment programme at later stages. For this reason, we sampled additional treatment cases from a list of new patients, i.e. all patients that have received treatment since the first sample was drawn, when conducting the four follow-up rounds of interviews with the cohort. Using six-monthly intervals, we sampled new interviewees randomly from the population of patients that had commenced treatment since the previous round of interviews. In the interest of affordability, the maximum number of new treatment cases to be sampled in each district in any one period equalled 40, which we believe would still allow us to generalise to the district and provincial ART programme levels in terms of the ART programme. This resulted in an additional 541 patients (*cross-sections*) being interviewed.

The current study employs both datasets (baseline cohort data and cross-sectional data) to provide an overview of the characteristics of the patients enrolling into the Free State public-sector ART programme during its first three years. For budgetary reasons, the 371 patients sampled in the above manner represented both the baseline of the cohort study (see 1^st ^objective) as well as the sample for the first representative cross-section of the ART programme (see 2^nd ^objective). The total sample size was thus 912 patients (371 cohort patients and 541 cross-sections) who started treatment between 2004 and 2006. These were all adult patients who were medically certified as ready for ART (CD4 < 200 cells/μL and/or WHO stage IV AIDS defining illness). Written, informed consent was obtained from all study participants by the nursing personnel at the respective clinics, as well as by the enumerators. In addition to the interview data, clinical data on the cohort patients (N = 371) were collected directly from the patient files, only after the written consent of all the patients had been obtained and with the authorisation of the provincial Department of Health.

### Data collection and study variables

To assess the demographic characteristics of the respondents, data were collected on sex, age, population group (black, coloured, white or Asian), and marital status (single/not living together, living together and unmarried, or living together and married). Our sample did not include patients from Asian origin, which is not surprising as recent estimates indicate that the Asian population only accounts for 0.1% of the Free State population [30].

Educational level and personal monthly income were added to the model as classical indicators of the patient's socio-economic position. To measure educational level, five educational categories were established based on the patient responses: no education, primary school, some secondary education, grade 12, and tertiary education. Employment status (employed, unemployed) was measured dichotomously. Because unemployment is high in South Africa (and subject to temporal changes), changes in the labour market participation rate (defined here as the percentage of the population of working age currently employed or actively seeking work) were assessed [31]. Personal monthly income (excluding social welfare grants) was included in the analysis, as a continuous variable. To further assess the socio-economic status of the participants, they were asked whether they received a social welfare grant (in particular, a disability grant). Four neo-material measures were included because previous analyses had shown them to be important determinants of socio-economic position [32-36]. The type of housing that the respondents occupied was categorised dichotomously into informal or traditional dwellings versus formal dwellings. A second variable measuring socio-economic status referred to the size of the dwelling: a continuous variable measured the number of rooms the household actively used. However, the absolute size of the dwelling is often an inaccurate measure of the actual space per occupant because the sizes of households can vary greatly. We therefore also considered the ratio of the dwelling size to the household size (i.e., the average number of rooms per person). Finally, the type of toilet facility (flush toilet, pit latrine, bucket latrine, chemical toilet, none) and its location (inside dwelling, on-site/in yard, off-site/outside yard) were used as neo-material indicators of the socio-economic positions of the respondents [32].

An obvious way of limiting access to treatment is to offer ART only to patients residing in specified geographic catchment areas [37-39]. The South African Department of Health did not use such an explicit geographic rationing strategy. However, the ART programme was to be phased in over a five-year period with the aim of achieving universal coverage of new AIDS cases by the end of the 2008/09 financial year. In the Free State, one service point in each of the five districts was envisaged within the first two years, with a steady expansion thereafter to all appropriate facilities (20 local municipal areas in total) [40]. The obvious rationing system here is that the majority of those patients not living within these few initial catchment areas would be excluded unless they were willing to relocate [41]. To assess these potential geographic rationing systems, we would ideally measure the Euclidian (or straight-line) distance between each patient's home and the specific assessment site where the patient accessed ART [29]. However, in the absence of such data, we used two proxy measures to evaluate the potential geographic differences among patients enrolling at different points in time. First, we investigated the proportion of patients who had to move, i.e., from one residence to another to access public-sector ART. Second, research in South Africa and Malawi identified transport costs as a barrier to the initial uptake of ART, as well as to patient adherence [42,43]. Therefore, it was useful to know how the cost to the patient, of transport to the ART facility, changed over time. In practice, the cost of a one-way trip to the ART facility was used to measure changes in this potential barrier to the uptake of and adherence to ART.

The medical selection criteria required that the patients were symptomatic (WHO clinical stage IV AIDS defining illness) and/or had a CD4 lymphocyte count below 200 cells/μL blood. A physician subsequently assessed the patient to confirm that the patient fulfilled the criteria for treatment and to exclude any reason for delaying treatment (e.g., untreated tuberculosis). The patients were then referred back to the assessment site for drug readiness training, a programme lasting three weeks [44]. There was no prioritisation for ART. The patients were randomly chosen for ART from those eligible for the programme and were not given preference based on CD4 count, viral load, or the time waiting for ART [23,45]. However, the demand for ART greatly surpasses its supply, so a study of the medical characteristics of those eligible patients actually accessing treatment is very useful [46]. CD4 cell counts and plasma HIV viral RNA levels, measured immediately before the commencement of ART, were used as the baseline measures of CD4 cells and viral load, respectively. These clinical data were only gathered from patients sampled for the first round of interviews (N = 371), which included only those patients who started treatment in 2004 and 2005. In the absence of clinical data from the patient files on all the study participants, two additional measures were used to assess the medical status of the patients enrolled in the public-sector ART programme: the time since their first diagnosis and their treatment status. Ample studies have shown that the delayed initiation of ART significantly reduces the chance of treatment success [47-49]. For this reason, the time since the first HIV-positive diagnosis was included in this study to assess the HIV-positive patient's trajectory towards ART. "Treatment status" distinguished treatment-naïve patients from patients who had previously received ART (from disease management programmes, workplace treatment programmes, or NGO programmes) before enrolling in the public programme.

### Data analysis

The differences in demographic (age, sex, population group and marital status), socio-economic (education, income, living circumstances), geographic (travel costs, relocation for ART), and medical (CD4, viral load, time since the first diagnosis, treatment status) of the various patient groups' (2004-2006) characteristics were assessed using one-way analysis of variance (ANOVA). An additional Bonferroni post-hoc analysis was used to assess the differences between the patient groups who enrolled at different times. Differences between the patient groups in nominal and ordinal variables were examined using cross-tabulations and were tested for statistical significance with a χ^2 ^test [50]. All analyses were performed with the statistical software package SPSS version 15.0.

## Results

### Sample description

The mean age of this sample of people living with AIDS was 37.4 years (Table 1). Of the 912 ART patients included in the study, 71.1% were women (Table 2). Descriptive statistics showed an over-representation of the black population group, whereas only a very small proportion of the participants were coloured or white. Our sample did not include patients from Asian origin, which is not surprising as recent estimates indicate that the Asian population only accounts for 0.1% of the Free State population [30]. Univariate analysis showed that 18.6% of respondents were married, whereas 9.7% of respondents lived together with a partner to whom they were not married. However, the majority of respondents did not cohabit with a partner (single, divorced, widowed, living apart). Education levels were relatively low, with only 17.1% of the sample (14.6% + 2.5%) having completed secondary education and only 2.5% having some form of tertiary education. The vast majority of respondents had only completed primary education or had started but did not finish secondary education, whereas 4.4% had no formal education at all. Overall, only 16.6% of respondents had worked for pay, profit, or family gain in the seven days preceding the interview. The mean personal monthly income of these employed respondents was ZAR1657.8 (SD = ZAR1411.9). The labour market participation rate indicated that 43.1% of respondents had either worked or taken active steps to find work in the four weeks before the interview. More than half the respondents received a social welfare grant (particularly a disability grant), which amounted to ZAR740, ZAR780, or ZAR820 per month, depending on when the interview was conducted. The large majority of respondents lived in a formal dwelling (76.7%), meaning that only 23.3% of respondents lived in an informal or traditional dwelling. The mean number of rooms used by the household was 3.8. The assessment of sanitary facilities showed that 70.4% of respondents had access to a flush toilet and almost all respondents (96.3%) had access to a toilet in either their house or yard. The mean cost incurred by a one-way trip to the facility was ZAR8.4, with 14.5% of patients incurring no cost to get to the facility. Only 1.1% of respondents had relocated to access public-sector ART. The respondents had been diagnosed with HIV from three days to 15 years before their entry into the programme (mean = 21.4 months). The mean CD4 cell count at baseline (ART initiation) was 120.8 cells/μL. At the start of treatment, the mean baseline viral load was 302,490 copies/mL. Finally, 6.6% of patients had previously received ART before the Free State public programme began.

**Table 1:** One-way ANOVA of the temporal differences in the demographic, socio-economic, geographic, and medical characteristics of patients enrolled in the Free State public-sector anti-retroviral treatment programme between 2004 and 2006 (N = 912)

	2004	2005	2006	Total	F scores
	(n = 223)	(n = 485)	(n = 204)		(df)
Age * (years, mean [SD])	37.9 (9.0)	37.7 (8.3)	36.1 (7.7)	37.4 (8.4)	3.192 (2, 844)

**Personal monthly income^a ^(ZAR, mean [SD])**	1774.2 (1439.0)	1737.6 (1486.8)	1007.9 (760.3)	1657.8 (1411.9)	1.727 (2, 108)

**Dwelling size, number of rooms^a ^(mean [SD])**	3.9 (1.9)	3.7 (1.6)	4.0 (1.7)	3.8 (1.7)	0.044 (2, 844)

Transport cost * (ZAR, mean)	6.8 (9.1)	8.5 (12.4)	10.1 (14.7)	8.4 (12.2)	3.259 (2, 589)

CD4 cell count at ART initiation *	109.3 (73.7)	138.9 (73.6)		120.8 (74.8)	4.229 (1, 109)

**Viral load at ART initiation^a^**	327113.4 (710803.5)	255867.3 (511759.9)		302490.1 (646032.4)	0.638 (1, 226)

Days since first HIV-positive diagnosis * (mean [SD])	772.7 (971.7)	625.4 (747.8)	545.9 (748.3)	642.9 (806.9)	3.756 (2, 820)

* p < .05

^a ^not significant (P ≥ 0.05)

**Table 2:** χ^2 ^test of the temporal differences in the demographic, socio-economic, geographic, and medical characteristics of patients enrolled in the Free State public-sector anti-retroviral treatment programme between 2004 and 2006 (N = 912)

		2004	2005	2006	Total	Significance
		(n = 223)	(n = 485)	(n = 204)		
**Sex**	Male	35.8	27.6	24.6	28.9	< 0.05
	Female	64.2	72.4	75.4	71.1	

**Population group**	Black	94.7	94.7	91.1	94.0	< 0.05
	Coloured	4.1	5.3	8.9	5.8	
	White	1,2	0.0	0.0	0.2	

**Marital status**	Married, living together	21.9	19.0	14.1	18.6	n.s.
	Unmarried, living together	7.3	10.3	10.5	9.7	
	No cohabitation with partner	70.8	70.7	75.4	71.7	

**Education**	No formal education	3.1	5.2	3.5	4.4	< 0.05
	Primary education	23.3	33.8	33.3	31.3	
	Some secondary education	52.3	45.4	46.8	47.2	
	Grade 12	15.5	14.0	15.2	14.6	
	Tertiary education	5.7	1.6	1.2	2.5	

**Work for pay**	No	80.8	85.4	80.7	83.4	n.s.
	Yes	19.2	14.6	19.3	16.6	

**Labour market participation**	No	63.5	57.9	46.1	56.9	< 0.005
	Yes	36.5	42.1	53.9	43.1	

**Social welfare grant**	No	47.7	43.2	46.2	44.8	n.s.
	Yes	52.3	56.8	53.8	55.2	

**Dwelling type**	Formal	77.7	74.2	82.5	76.7	n.s.
	Informal/traditional	22.3	25.8	17.5	23.3	

**Toilet type**	Flush toilet	60.1	68.7	87.1	70.4	< 0.001
	Pit latrine	30.1	20.8	11.2	21.0	
	Bucket latrine	8.8	9.1	1.8	7.5	
	Chemical toilet	0.5	1.0	0.0	0.7	
	None	0.5	0.4	0.0	0.4	

**Toilet site**	Inside dwelling	28	31.5	49.4	34.3	< 0.001
	On-site/in yard	69.4	63.5	49.4	62.0	
	Off-site/outside yard	2.6	4.9	1.2	3.7	

**Moved for ART**	No	98.4	99.0	99.4	98.9	n.s.
	Yes	1.6	1.0	0.6	1.1	

**Previous ART**	No	88.6	94.0	97.0	93.4	< 0.005
	Yes	11.4	6.0	3.0	6.6	

n.s. not significant (P ≥ 0.05)

### Differences in socio-demographic characteristics across patient groups

We used cross-tabulation with the χ^2 ^test to examine the association between the patient's sex and the year in which he/she enrolled in the Free State public-sector ART programme. The analysis showed a significant association between the patient's sex and the year of enrolment. In 2004, 64.2% of the new patients were female, and this proportion increased steadily to 75.4% in 2006. One-way ANOVA was used to assess the association between patient age and the year of enrolment. When the results of the ANOVA were statistically significant (*P *< 0.05), post-hoc Bonferroni multiple comparisons were made to determine where statistically significant differences between the group means existed (Table 3). The analysis revealed significant differences in patient age across enrolment years. Further post-hoc analyses revealed that the ages of the patient groups were only significantly different between the first two and the third year of the ART programme, indicating that the mean age of the patients who started ART in 2004 was similar to that of the patients who started ART during the following year, whereas the patients who started in 2006 were slightly, but significantly, younger than those who started treatment during the previous two years. A bivariate analysis using the χ^2 ^test revealed no significant association between the patient's marital status and the year of enrolment. However, population group (black, coloured, or white) was significantly associated with the year of enrolment. The study findings show a steady increase in the proportion of coloured patients enrolling in the programme, while the proportion of black patients decreased slightly. However, the latter remained the dominant population group in the programme.

**Table 3:** Pairwise comparisons: Bonferroni tests for differences in mean age, transport cost, and days since the first HIV-positive diagnosis over time (N = 912)

Multiple comparisons	Age	Transport cost	Days since first diagnosis
	**Difference**	**Difference**	**Difference**

**2004-2005**	0.138^a^	-1.758*	147.300*
**2004-2006**	1.900*	-3.399*	226.828**
**2005-2006**	1.762*	-1.641^a^	79.497*

* p < .05, ** p < .01

^a ^not significant (P ≥ 0.05)

### Differences in socio-economic status across patient groups

First, the analysis revealed significant differences in the patients' educational levels across the first three years of ART enrolment (*P *< 0.05). The proportion of patients who had not undertaken at least some secondary education rose from 26.4% in 2004 to 39.0% in 2005. In 2006, no further increase was observed. A bivariate analysis showed no significant association between employment status and the year of enrolment in the ART programme. If we focus on the employed patients, one-way ANOVA revealed no significant differences between the patients' personal monthly incomes across the first three years of ART enrolment. A bivariate analysis suggested that the labour market participation rate among patients enrolled in the Free State public ART programme steadily increased during the first three years of the programme. Initially, only 36.5% of patients were either employed or actively looking for employment; this proportion rose to 42.1% in 2005 and to 53.9% in 2006. Finally, we investigated those patients who were unable to work: cross-tabulation with a χ^2 ^test indicated that the proportion of enrolled patients who received a disability grant did not change significantly over time.

Second, this study included four neo-material indicators of socio-economic position to assess any potential differences between the patients enrolled at different stages of the ART programme. A bivariate analysis using a χ^2 ^test showed that there was no significant association between dwelling type and the year of enrolment. Throughout the study period, approximately four of every five respondents lived in a formal dwelling, and the remainder lived in an informal or traditional dwelling. One-way ANOVA revealed no between-group differences with regard to the size of the dwelling, making post-hoc Bonferroni multiple comparisons redundant. The study results showed that the size of the dwelling remained stable between 2004 and 2006. However, the absolute size of the dwelling is often an inaccurate measure of the actual space per occupant because the sizes of households can vary greatly. When we considered the ratio of the dwelling size to the household size (i.e., the average number of rooms per person), one-way ANOVA again revealed no significant between-group differences, indicating that the ratio of dwelling to household size did not differ significantly across the initial years of the public anti-retroviral programme. The final two neo-material indicators of the patients' socio-economic status assessed the household's sanitary facilities. The study results showed a steady and significant increase in the proportion of patients with access to a flush toilet. Consequently, the proportion of households living under poor sanitary conditions decreased over time. A similar trend was apparent when the locations of these toilet facilities were assessed: in 2004, less than one third of the patients enrolled in the ART programme had a toilet inside their dwelling; in 2006, this proportion had increased to 49.4%.

### Geographic differences across patient groups

We attempted to assess whether the Free State's phased approach to ART implementation had an unintended impact on who enrolled in the public-sector ART programme. The initially small number of service points in each of the five districts could have favoured patients living within the immediate catchment areas of the facilities. One-way ANOVA was used to assess the association between the patient's transport costs and the year of enrolment, and showed significant temporal changes in the mean cost of a one-way trip to the facility. The post-hoc Bonferroni test indicated that the mean cost of a one-way trip to the ART facility was significantly lower in 2004 than in the two subsequent years (Table 3). There was no significant difference in the travel costs in 2005 and 2006. However, it is possible that those patients not living within the few initial catchment areas had relocated to live near the ART facility. Consequently, we also assessed the association between year of enrolment and whether the patient had relocated to access public-sector ART. A bivariate analysis revealed no significant association: overall, very few patients had ever relocated to access ART and this percentage remained stable throughout the study period.

### Medical differences across patient groups

Due to the fact that the number of patients medically eligible for ART greatly exceeds the number of patients actually accessing the treatment, in this study, we wanted to investigate the trajectory that patients complete between their first HIV-positive diagnosis and their enrolment in the public-sector ART programme. First, the time since the first HIV-positive diagnosis was assessed. One-way ANOVA was used to assess the association between the time since their first diagnosis and the year of enrolment. The analysis revealed that the delay between the first HIV-positive diagnosis and the start of public-sector ART differed significantly across the first three years of the ART programme. To determine exactly where these differences occurred, we examined the results of Bonferroni multiple comparisons (Table 3). The post-hoc tests revealed that the time since the first HIV-positive diagnosis steadily and significantly declined throughout the entire study period. In 2004, a patient knew that he/she was HIV positive for, on average, 772.7 days (SD = 971.7) before starting ART; in 2005, the mean period was 625.4 days (SD = 747.8; *P *< 0.05); and in 2006, this period had decreased to 545.9 days (SD = 748.2; *P *< 0.05). Second, we analysed the patients' trajectories towards public-sector ART by investigating whether the patients had received ART from an alternative provider (disease management programmes, workplace treatment programmes, or NGO programmes) before enrolling in the public-sector ART programme. A bivariate analysis showed a significant association between treatment status (treatment naive or not) and year of enrolment. The patients who enrolled in 2004 (11.4%) were almost four times more likely to have previously received ART from an alternative provider than were patients who commenced public-sector ART in 2006 (3.0%), showing a clear trend in the treatment status of enrolling patients over time. Third, one-way ANOVA revealed a significant difference in the CD4 cell counts at the beginning of ART of patients who started treatment in 2004 and those who started in 2005. The mean number of CD4 cells/μL blood at the start of ART increased from 109.3 (SD = 73.7) in 2004 to 138.9 (SD = 73.6) in 2005. Finally, our analysis showed no significant association between the baseline viral load and the year of enrolment.

## Discussion

During the introductory years of the South African public-sector ART programme (2004-2006), anti-retroviral coverage rose from 4.9% in 2004 to 19.1% in 2006 (and to 40.2% in 2008). The preliminary patient outcomes of the programme were positive [4,49], indicating that South Africa ranks among the top African countries for patient survival and retention rates, approaching those reported in industrialised countries [11,12]. Despite significant progress in the provision of ART in South Africa, human and financial resource shortages create a large gap between the supply and demand for ART. Currently, only four of every 10 South Africans requiring treatment are actually receiving it. The situation is most acute in the Free State, where ART coverage is only 25.8% [19]. Against the backdrop of this gap between supply and demand, Stewart et al. (2006) raised concerns that the provision of ART will create and/or exaggerate inequities in service provision [51]. Therefore, it is crucial to monitor who gains access to these life-saving drugs when anti-retroviral programmes are implemented in regions with high HIV prevalence and limited resources [26]. Using Rosen's typology of implicit rationing systems [25], we measured the demographic, socio-economic, geographic, and medical characteristics of the patients enrolling in the Free State public-sector ART programme between 2004 and 2006.

The study results revealed significant differences in all four domains. Demographically, the results showed a significant increase over time in the proportion of females entering the programme, resulting in an under-representation of male patients in the public-sector ART programme. A similar trend was found in an Ethiopian study by Kloos et al. (2007) and a Thai study by Le Cœur et al. [52,53]. Because HIV infection rates are higher among women, it is to be expected that more women than men are receiving ART. However, our findings show that the proportion of women undergoing treatment was significantly higher than would be expected given the demographic trends [54]. In 2006, three of every four (75.6%) patients enrolling in the ART programme were female, whereas the expected percentage based on national reports was only 56% [55]. A WHO report on the global access to anti-retroviral therapy [55] speculated that the relative over-representation of women in ART programmes could be because women are better integrated into community networks, and thus have better health-care information and are better able to access public health facilities than are men, especially where these women access PMTCT programmes. Closely related, recent studies suggest that antenatal clinics are often the first point of entry into HIV/AIDS prevention, care and treatment [56,57]. Nattrass (2008) hypothesised that South African men with AIDS-related illnesses are less likely to access ART than their female counterparts because masculine norms encourage them to deny weakness and to avoid seeking treatment for any ailment for as long as possible [54]. Another possible explanation for this difference is the fact that the South African employment rate is substantially higher among males (50.5%) than among females (34.0%) [58]. Because each visit to the clinic can be assumed to require a full day off work as a result of the protracted waiting times at the facilities [14,59], the cost of losing a day of work for health care may still be too high [60,61], resulting in the reported under-representation of employed (predominantly male) patients. However, further research is required to fully disentangle the complex relationship between sex and public-sector ART access. Our study also revealed significant but very small differences in the ages of the patients enrolling at different time points, with more recent patients being slightly younger. However, this very small reduction in the mean age of treatment initiation does not warrant any policy measures. Finally, the proportion of coloured patients accessing public-sector ART increased over time, although black patients still constituted the largest population group in the programme. These findings confirm those of previous studies performed by Natrass (2006), Tladi (2006), and Hudspeth (2004) [62-64].

As we would expect in a public-sector health-care programme, the ART patients can be described as relatively poor in certain respects: only one of every five respondents was employed and this percentage remained stable over time. Analysis of the association between socio-economic status and access to public-sector ART revealed a growing trend in labour-market participation among ART patients over time. In other words, patients enrolling in 2006 were more likely to be either employed or looking for work than were patients enrolling during the first two years of the programme. However, our analysis of employment trends displayed no significant direction, indicating that the proportion of employed patients remained low. As suggested above, one potential explanation is that employed (predominantly male) patients are under-represented in the ART population because of the protracted waiting times at the facilities, warranting further research on this topic. Our study found no significant association between the remaining classical indicators of socio-economic status (monthly income, disability grant, employment status) and entry into the ART programme. The analysis of neo-material indices of health inequality showed a clear trend in the housing conditions of the enrolled patients over time: the toilet facilities (both their place and type) improved significantly during the three-year study period. Previous studies by Anderson et al. (2006) and Booysen et al. (2006 and 2007) have already shown that that the initial group of ART patients was not the poorest of the poor [29,65,66]. Our finding that the majority of patients had access to a flush toilet, either in their dwelling or their yard, confirmed these conclusions and also suggests that these inequities worsened over time.

In previous studies, the distance from home to the health facility was often cited as one of the most important barriers to the initial uptake of ART and to patient adherence [42,67]. Our study showed that the mean transport cost of a one-way trip to the facility increased significantly (from ZAR 6.8 to ZAR 10.1) over the first three years of the Free State public-sector ART programme. A potential explanation would be that the initial (most urgent) treatment patients moved to live close to the ART facility, but the study results showed no significant association between the year of enrolment and whether the patient had relocated to access ART. It is also noteworthy that the intention of the Free State was that the programme implementation would occur in a phased or staggered manner, and as resources and experience became available [44]. During phase I of the ART roll-out in the province (May-December 2004), four treatment sites, 13 assessment sites, and three combined sites were established. In phase II (2005/2006), a further three treatment, eight assessment, and seven combined sites were introduced [68]. Because of this gradual establishment of more ART facilities, it would be logical to see an accompanying gradual reduction in the mean distance (and transport cost) to the nearest ART facility. The evidence suggesting that cost is an important barrier to ART uptake and adherence indicates that further research is required to investigate this worrying trend.

Finally, our study results reveal that eligible patients (CD4 < 200 cells/μL and/or WHO stage IV AIDS) are gradually enrolling at a slightly earlier stage of HIV infection. The interval between the first HIV-positive diagnosis and ART initiation is steadily decreasing over time. This is also reflected in the increasing baseline CD4 cell counts at ART initiation. This trend towards earlier enrolment and initiation of treatment is potentially important because many studies have indicated that delayed ART initiation (low baseline CD4 and high viral load) significantly reduces the chance of ART success [21,69]. These results for the initial years of public-sector ART are also consistent with the recommendations for ART scale-up made by Walensky et al. (2008) [23]. Their simulation model showed that the prioritisation of the sickest patients (i.e., those with the lowest CD4 counts) would lead to significantly fewer deaths compared with the number of deaths in non-prioritised scenarios. However, this positive trend does not justify complacency. The overall low baseline CD4 cell count among the ART patients (120.8 cells/μL) in our sample is similar to that observed in previous studies in South Africa [35,70,71]. These results once again show that increasing the recommended CD4 count at which patients begin ART from 200 to 350 cells/μL blood, as stated in the new WHO protocol for ART, will require extensive efforts to reach these currently untreated patients [72]. This will prove to be a difficult task in the context of crippling human and financial resource shortages for health services [17]. Finally, the study showed a clear trend of more ART-naïve patients being enrolled in the public-sector ART programme over time, whereas earlier patients had received ART from other sources before transitioning into the programme. The introduction of free public-sector ART clearly attracted patients from alternative ART programmes, while gradually eligible patients increasingly directly enrolled into the public-sector programme.

The strengths of this study include its longitudinal character and the availability of information on an understudied population. To the best of our knowledge, this is one of the first studies to assess, in a quantitative manner, the demographic, socio-economic, geographic, and medical characteristics of a representative sample of HIV-infected patients enrolling in South Africa's public-sector ART programme [52]. However, there are some limitations to our study. First, our data set only includes eligible patients enrolling before 2007. Further research is required to monitor the more recent trends in patient characteristics. Second, the study only included patients eligible for ART who actually presented for and accessed treatment. As stated before, the Free State's anti-retroviral coverage was only 25.8% in 2008 [19]. Consequently, further research is required to investigate the characteristics of the most vulnerable HIV/AIDS patients, those eligible for treatment but who do not access it. Third, a comparatively small number of variables, based on the available survey items, was used to measure the demographic and medical characteristics of the patients, potentially restricting the reliability and validity of these measures. Finally, the reported associations between the patient characteristics and their entry into the ART programme may not be generalisable to alternative settings. We can only ascribe the findings to patients enrolled in a public-sector ART programme and, more specifically, to patients enrolled in South Africa's public ART programme, as implemented in the Free State Province.

## Conclusions

Our analysis of 912 HIV/AIDS patients starting ART in the Free State public-sector ART programme showed significant changes in the demographic, socio-economic, geographic, and medical characteristics of the patients during the first three years of the programme. The patients accessing treatment tended to be female and unemployed. Although relatively poor, those who were most likely to access ART services were not the most impoverished social group. The proportion of female clients increased and their socio-economic situation improved between 2004 and 2006. The increasing mean transport cost for a visit to the facility is worrying, because this cost is an important barrier to ART uptake and adherence. Eligible patients are accessing ART at an increasingly earlier stage of HIV infection, which is a positive trend because delayed ART initiation significantly reduces the chance of ART success. Although our knowledge of the characteristics of those patients actually accessing public-sector ART in a resource-poor setting is still in its infancy, this analysis has both practical and theoretical implications.

Theoretically, this study contributes to our understanding of the impact of a large-scale public ART programme on the population of patients eligible for treatment. The study results show that as long as the demand for ART exceeds the supply, implicit rationing of anti-retroviral therapy for HIV/AIDS is inevitable. Despite national policies that recognise the importance of equitable access to ART, the early evidence in the Free State is that these processes can have unwanted inequitable effects. The research community should devote more attention to the potential unintended consequences of ART scale-up in the context of large patient numbers and limited human and financial resources.

In practical terms, the study results show the importance of monitoring the population with access to ART programmes in the regions where they are most needed [26]. A knowledge of the characteristics of the public-sector ART clients can potentially facilitate the successful implementation of the *NSP*. To reach the *NSP*'s ambitious goal of providing treatment to 80% of HIV/AIDS patients who require it by 2011 [3], policy makers need data from research studies about the eligible patients who are accessing ART and those who are not, to maximise the policy outcomes in a context of limited resources. A knowledge of the characteristics of the enrolled patients can assist policy makers in developing measures to retain these patients in care, a *conditio sine qua non *for every ART programme. Moreover, the information provided can be used to identify and target patient groups that are not currently benefitting from the implementation of the ART programme.

## Competing interests

The authors declare that they have no competing interests.

## Authors' contributions

EW participated in the design of the study, performed the statistical analysis, and wrote the manuscript. KP and FB gave advice on interpreting the results. CH, FVL, DvR and HM were involved in revising the article for important intellectual content. All authors read and approved the final manuscript.

## Pre-publication history

The pre-publication history for this paper can be accessed here:

http://www.biomedcentral.com/1471-2458/10/387/prepub
